# Measuring the financial impact of disabilities in India (an analysis of national sample survey data)

**DOI:** 10.1371/journal.pone.0292592

**Published:** 2023-10-12

**Authors:** Jeetendra Yadav, Niharika Tripathi, Geetha R. Menon, Saritha Nair, Jitenkumar Singh, Ravinder Singh, M. Vishnu Vardhana Rao

**Affiliations:** 1 ICMR-National Institute of Medical Statistics (NIMS), New Delhi, India; 2 Department of Sociology, Indraprastha College for Women, University of Delhi, New Delhi, India; 3 Indian Council of Medical Research (ICMR), New Delhi, India; Indian Institute of Dalit Studies (IIDS), INDIA

## Abstract

**Background:**

People with disabilities are vulnerable because of the many challenges they face attitudinal, physical, and financial. The National Policy for Persons with Disabilities (2006) recognizes that Persons with Disabilities are valuable human resources for the country and seeks to create an environment that provides equal opportunities, and protection of their rights, and full. There are limited studies on health care burden due to disabilities of various types.

**Aim:**

The present study examines the socioeconomic and state-wise differences in the prevalence of disabilities and related household financial burden in India.

**Methods:**

Data for this study was obtained from the National Sample Survey (NSS), 76th round Persons with Disabilities in India Survey 2018. The survey covered a sample of 1,18,152 households, 5,76,569 individuals, of which 1,06,894 of had any disability. This study performed descriptive statistics, and bivariate estimates.

**Results:**

The finding of the analysis showed that prevalence of disability of any kind was 22 persons per 1000. Around, one-fifth (20.32%) of the household’s monthly consumption expenditure was spent on out-of-pocket expenditure for disability. More than half (57.1%) of the households were pushed to catastrophic health expenditure due to one of the members being disabled. Almost one-fifth (19.1%) of the households who were above the poverty line before one of members was treated for disability were pushed below the poverty line after the expenditure of the treatment and average percentage shortfall in income from the poverty line was 11.0 percent due to disability treatment care expenditure.

**Conclusion:**

The study provides an insight on the socioeconomic differentials in out-of-pocket expenditure, catastrophic expenditure for treatment of any kind of disability. To attain SDG goal 3 that advocates healthy life and promote well-being for all at all ages, there is a need to recognize the disadvantaged and due to disability.

## Background of the study

*People with disabilities are vulnerable because of the many barriers we face attitudinal, physical, and financial. Addressing these barriers is within our reach and we have a moral duty to do so. But most important, addressing these barriers will unlock the potential of so many people with so much to contribute to the world*. *Governments everywhere can no longer overlook the hundreds of millions of people with disabilities who are denied access to health, rehabilitation, support, education, and employment and never get the chance to shine” -**Stephen Hawking.***

According to CDC disability is any condition of the body or mind (impairment) that makes it more difficult for the person with the condition to do certain activities (activity limitation) and interact with the world around them (participation restriction). The World Health Organization (WHO) recognizes three dimensions of disability viz. Impairment, activity limitation and participation restriction. According to the World Health Organisation, 15% of the world’s population experiences one or the other form of disability. According to government of India there were eight categories of disability were namely viz (i) seeing disability, (ii) hearing disability, (iii) speech disability, (iv) movement disability, (v) mental retardation, (vi) mental illness, (vii) any other disability, and (viii) multiple disabilities [1, 2]. The categories of mental retardation, mental illness, and any other disability were used for the first time. According to the Census 2011, there are 2.68 Crore Persons with Disabilities (PwDs) in India constituting 2.21% of the total population. The percentage of disabled in the total population increased from 2.13% in 2001 to 2.21% in 2011. In rural areas, the increase was from 2.21% in 2001 to 2.24% in 2011 whereas, in urban areas, it increased from 1.93% to 2.17% during this period [3–6]. The World Bank figures states that there are more than a billion people with disabilities among which 80 percent are in developing economies such as India. Although having a disability is often associated with severe socioeconomic disadvantages and poverty, only a small fraction of the people with disabilities in India receive government assistance [7, 8].

Disability imposes several kinds of economic burdens on the disabled individual, on the family of the disabled, and more broadly on the employer, organizations, and society. The disability of a family member affects the family’s standard of living. While the disabled person has to face the conversion handicap as described by Amartya Sen (2005) to maintain a standard of living equivalent to a normal individual, he also loses productivity due to loss in workdays and due to the disability, itself. The other members of the household who are caregivers are forced to reduce their working hours thus reducing the overall family income. Thus, a disability in the household results in dual challenges; increase in household consumption due to conversion handicaps and decrease in household consumption due to fall in the family resources. Not much is known about the magnitude of the medical expenses of disability in developing countries. In developed countries, a few studies estimate medical costs and loss in earnings due to some disabilities. Several previous studies [9–15] have demonstrated measurement issues in this area. No studies have been done in India on disabilities and their financial burden.

## Rationale of the study

Persons living with disabilities excessively suffer from poor health and poverty. As per authors knowledge and literature review, there is paucity of literature that talk about financial impact of disability for this helpless subpopulation, mainly in countries like India. Therefore, there is a need for analysis of financial burden of disability, and it will help policy makers and programmers to design health systems for disabled persons. Based on the above literature, this study pursues to fill this gap and aims to examine the prevalence of different types of disabilities and related financial burden for treating the different types of disabilities in India.

## Data and methodology

### Data sources

This study used the data from the countrywide NSS, 76th round which was based on the theme, Persons with Disabilities in India Survey conducted during July-December 2018. The NSS surveys were done by the Ministry of Statistics, Planning, and Implementation (MOSPI), India, and covered all the states of India [16]. The findings of NSS surveys are widely used for policy construction by experts, researchers, and governments.

### Sampling design and sample size

The NSS 76^th^ round survey implemented a stratified two-stage sampling design using Census 2011 as a sampling frame. In the first stage of the sampling villages/urban blocks and in the second stage households in both rural and urban areas were selected. The survey covered all the states and union territories of India. NSS76^th^ round covered 8992 village/urban blocks (5378 rural village and 3614 urban blocks), covering 1,18,152 households (81004 in rural and 37148 in urban) enumerating 576,569 individuals (4,02,589 in rural and 1,73,980 in urban). The present study contains of a total 1, 06,894 persons (74,946 in rural, 31,948 in urban, 61,567 males, and 45,305 females) who reported at least one disability during the survey.

## Outcome indicators

### Disability

The major types of disability studied in this paper are discussed in Table 1.

**Table 1:** Types of disability and their description.

S.NO	Type of Disability	Description
1	Locomotor disability	Acid attack victims, leprosy cured person, polio, cerebral palsy, dwarfism, muscular dystrophy and other locomotor disability
2	Visual disability	Blindness and low vision
3	Hearing disability	Hearing disability
4	Speech and language disability	Speech and language disability
5	Mental retardation/intellectual disability	Specific learning disabilities, autism spectrum disorder and other mental retardation/ intellectual disability
6	Mental illness	Mental illness
7	Other disabilities	Chronic neurological conditions, parkinson’s disease, multiple sclerosis, other chronic neurological conditions, blood disorder, thalassemia, haemophilia and sickle cell disease

**Source**: NSS report Persons with Disabilities in India, NSS 76th round (July-December 2018)

### Average out-of-pocket expenditure

Out-of-pocket expenditure (OOPE) was calculated as the sum of direct medical costs (i.e., hospital stay, consultation, treatment medicines and procedures, laboratory, and other investigation charges), and direct non-medical costs (i.e., transportation, meals, lodging: for patients and care givers). The NSS 76th round survey captured both the medical expenditure (ME) (surgery, equipment, hospitalization, etc.) and nonmedical expenditure (NME) (transport, lodging, food, etc.) for infrequent expenditure during last 365 days and usual monthly medical expenditure (UMME) (excluding those infrequent medical expenditure covered during last 365 days) [17] (. For this study, the average out-of-pocket expenditure was calculated using the following procedure:

$$Average\;OOPE=\frac{\frac{ME+NME\;in\;last\;365\;days}{12}+UMME+UMNME}{PWD}$$

Where ME = Medical expenditure, NME = non-medical expenditure, UMME = usual monthly medical expenditure and UMNME = usual monthly non-medical expenditure, PWD = no. of persons who reported expenditure for their disability

### Average household monthly consumption expenditure (HCE)

It is a preferred measure since it is lesser prone to disparity and has less chance of being underreported or overreported when compared to income [18]. We have used average monthly household consumption expenditure (HCE) as a proxy variable for household income which was used in several previous studies [18, 19]. Based on the NSSO 76th round we calculated the average monthly consumer expenditure by [17].


$$HCE=\frac{A+B+C+\frac{D}{12}}{Total\;no.households}$$

Where HCE = Household consumption expenditure

A = usual consumer expenditure in a month for household purposes out of purchase, B = imputed value of usual consumption in a month from home grown stock, C = imputed value of usual consumption in a month from wages in kind, free collection, gifts, etc, and D = expenditure on purchase of household durables during last 365 days

### Share of out-of-pocket expenditure on total household expenditure

Share of out-of-pocket expenditure on total household expenditure for the ith household is defined as the percentage yearly share of OOPE in the yearly household consumption expenditure (HCE) [20–23] i.e.,

$$Share\;of\;out-of-pocket\;expenditure\;on\;total\;household\;expenditure=\frac{OOPE\;x\;100}{HCE}$$

Where HCE = Household consumption expenditure, OOPE = Out—of—pocket expenditure

### Catastrophic health expenditure (CHE)

Catastrophic spending on health occurs when a household reduces its basic expenses over a certain period of time, sells assets, or accumulates debts in order to cope with the medical bills of one or more of its members who are being treated for any disability. A household experiences catastrophic health expenditure (CHE) if the health care payment exceeds a threshold (x%) of the total household consumption expenditure ie OOPE>x% HCE or Share of out-of-pocket expenditure on total household expenditure >x%. Various studies have stipulated this threshold based on various theories [20, 24–26]. This study has used two approaches to determine the CHE.

#### First approach

In the first approach we have used two thresholds of 10% and 20% respectively i.e., when Share of out-of-pocket expenditure on total household expenditure >10% or Share of out-of-pocket expenditure on total household expenditure >20% based on the previous published studies [20, 27–31]. Thus the CHE definition for the ith household in the first approach

CHEi = 1 if Share of out-of-pocket expenditure on total household expenditure i>10%
CHEi = 0 if Share of out-of-pocket expenditure on total household expenditure i≤10%.
CHEi = 1 if Share of out-of-pocket expenditure on total household expenditure i>20%
CHEi = 0 if Share of out-of-pocket expenditure on total household expenditure i≤20%

where CHEi = 1 means the ith household experiences CHE and CHEi = 0 means the ith household does not exhibit CHE.

#### Second approach

In the second approach a household exhibits catastrophic health expenditure if the annual OOPE exceeds the average consumption expenditure of a household member ie CHE = 1 if OOPE >HCE/n where n is the number of members in the household. i.e a household is pushed to CHE when the out-of-pocket expenditure exceeds the average consumption expenditure of a household member.

We can also define CHE = 1 if OOPE>2HCE/n i.e a household is pushed to CHE when the out-of-pocket expenditure exceeds the consumption expenditure of two household members

We adopted this approach from a previous paper which argued that categorizing a household as experiencing CHE based on the first approach doesn’t reflect the true situation [17, 20, 32]. Instead, a household may be driven to CHE if its OOPE exceeds the average consumption of one or two household members.

### Poverty impact due to OOPE

Poverty was measured using two indices namely Poverty Head Count Ratio (PHCR) [27, 31] which is the percentage of households that fall below the poverty line (PL) due to OOPE and the Poverty Gap Ratio (PGR) [20, 21, 30] measured as the average percentage deficit from the poverty line due to OOPE [20, 21, 29, 31, 33]. For the present study we used PL = 1,407 INR, in urban areas and 972 INR, in rural areas as per the recommendation of the Rangarajan committee in 2014, based on the 2012 data [34]. Further, the PL for 2012 was adjusted for inflation using the consumer price index for December 2018 to obtain PL for December 2018 [35, 36].

A household which was above the poverty line is defined as impoverished if it falls below poverty line after incurring the healthcare expenditure i.e.,

Povertyheadcount(PHC)=1ifHCE≥PL&(HCE−OOPE)<PL

Otherwise, 0, where HCE is total yearly consumption expenditure of a household.

The poverty head count ratio (PHCR) is the percentage of households which fall below the poverty line due to OOPE i.e.,

$$Poverty\;head\;count\;ratio(PHCR)=\frac{\sum_{i=1}^{N}{Number\;of\;impoverished\;households}_{i}}{Total\;households\;(N)}\times 100$$

The percentage deficit from the poverty line of an impoverished household (PHC = 1) is quantified using the poverty gap (PG) i.e.,

$$Povery\;gap\;(PG)=PHC\times \frac{\left\{PL-(HCE-OOPE)\right\}}{PL}$$

The poverty gap ratio (PGR) measures the poverty gap of all impoverished households as a percentage of total households in the population.


$$Poverty\;gap\;ratio\;(PGR)=\frac{1}{Total\;HH\;(N)}\;\sum_{i=1}^{N}{Poverty\;gap}_{i}\times 100$$

### Socioeconomic variables

To study the financial burden of households due to disability, the present study included individual characteristics of the disabled person (age, sex, education, marital status), household characteristics (religion, caste, living arrangement, economic status, relation to head of the household) and community variable (place of residence and geographical region) based on the literature review and availability of data [37, 38].

### Statistical methods

Firstly, we determined the prevalence of different types of disability. Secondly, OOPE, CHE and impoverishment effect due to disability were analysed. Analysis was done on STATA 13.1 software [39]. Sampling weights with clustering were already calculated in the dataset. The details of sampling weight have been described in the NSS 76^th^ round report [16]. We used SVY command while using sampling weights [40]. All expenditures are reported in Indian Rupees (INR).

### Ethics approval

This study used the data from National Sample Survey (NSS), 76th round Persons with Disabilities in India Survey 2018. First, the NSS survey obtained the ethical consent from the Institutional review committee before the survey. Second, during the survey a usual taken written consent by the respondent. Therefore, no ethical approval is required separately for this study.

## Results

### Prevalence of disabilities

The prevalence of any disability was found to be 22 per 1000 persons in India with hearing impairment being most prevalent (2.2) followed by visual and speech disability. Among the disabled persons, 12.4 per 1000 person were found to be suffering from locomotor disability. Mental retardation and mental illness were found to be among 1.6 and 1.4 per 1000 persons. Irrespective of its type, disability was found to be higher among people who were aged 60 years and above, than those who were younger (Table 2). The prevalence of locomotor disability was highest in Punjab (18 persons per 1000 while visual disability was found to be highest in Sikkim (5.3 person per 1000), and mental retardation and mental illness was found to be highest in the State of Kerala (Table 3).

**Table 2:** Socioeconomic differentials in prevalence of disabilities per 1000 population.

Background Characteristics	Locomotor	Visual	Hearing	Speech and language	Mental retardation/ Intellectual disability	Mental illness	Other	Any disability
**Age (in years)**								
0–14	3.8	0.6	0.4	2.2	1.9	0.5	0.4	9.8
15–35	7.3	0.8	0.6	1.7	2.0	1.2	0.4	14.0
36–59	15.4	2.1	2.0	1.5	1.0	1.8	0.5	24.3
60 and above	49.2	12.3	14.8	2.2	0.6	2.7	1.7	83.7
**Gender**								
Male	14.0	2.1	2.2	2.1	1.8	1.4	0.6	24.3
Female	10.6	2.2	2.3	1.6	1.2	1.2	0.5	19.5
**Education**								
Illiterate	19.8	5.0	4.8	3.6	4.0	2.4	0.7	40.3
Up to Primary	9.9	1.4	1.6	1.8	1.1	1.0	0.5	17.5
Middle	11.1	1.2	1.4	1.1	0.6	1.2	0.5	17.3
Secondary and above	8.8	0.8	0.9	0.6	0.2	0.6	0.4	12.4
**Marital Status**								
Never married	6.4	1.0	0.6	2.6	3.0	1.3	0.5	15.5
Currently married	14.2	2.1	2.4	1.1	0.3	0.9	0.5	21.5
Others	44.6	12.3	14.2	2.6	1.1	4.3	1.2	80.4
**Relation to head of the household**								
Self/ Spouse of head	17.7	2.9	3.4	1.2	0.4	1.1	0.6	27.3
Married Child and spouse of child	6.4	0.5	0.5	1.2	0.4	1.0	0.3	10.4
Unmarried Child	6.5	1.0	0.6	2.6	3.1	1.4	0.5	15.8
Grand Child	2.9	0.3	0.3	1.6	1.7	0.3	0.4	7.6
Others family members and relatives	37.3	10.4	11.1	3.9	3.3	5.0	1.1	72.1
**Religion**								
Hindu	12.6	2.2	2.3	1.8	1.5	1.2	0.5	22.2
Muslim	10.6	1.8	1.6	1.9	1.7	1.7	0.5	19.8
Others	13.7	1.9	2.5	2.1	1.7	1.8	0.7	24.4
**Social group **								
SC/ST	13.0	2.4	2.3	2.1	1.5	1.3	0.6	23.1
OBC	12.1	2.2	2.2	1.8	1.6	1.3	0.5	21.7
Others	12.1	1.8	2.1	1.7	1.6	1.3	0.6	21.2
**MPCE quintile **								
Poorest	11.8	2.3	2.0	2.1	1.4	1.3	0.4	21.2
Poorer	12.4	2.4	2.2	2.1	1.6	1.4	0.4	22.5
Middle	12.8	2.4	2.5	2.0	1.6	1.4	0.5	23.2
Richer	12.8	2.2	2.2	1.9	1.7	1.3	0.6	22.7
Richest	12.1	1.6	2.1	1.4	1.5	1.2	0.8	20.6
**Place of residence **								
Rural	12.8	2.4	2.4	2.0	1.6	1.4	0.5	23.0
Urban	11.4	1.7	1.8	1.6	1.5	1.1	0.7	19.7
**Geographic region **								
North	13.8	2.1	2.2	1.5	1.6	1.4	0.3	22.9
Central	13.7	2.2	2.1	1.7	1.4	1.3	0.4	22.8
East	11.1	2.1	2.1	2.3	1.5	1.5	0.7	21.3
Northeast	7.0	2.6	2.5	1.8	0.8	0.9	0.3	16.0
West	11.6	1.6	1.7	1.5	1.7	1.0	0.7	19.9
South	13.0	2.4	2.9	2.1	1.9	1.3	0.7	24.4
Union Territories	7.9	1.0	1.2	0.9	1.1	0.9	0.3	13.4
**Total**	**12.4**	**2.1**	**2.2**	**1.9**	**1.6**	**1.3**	**0.5**	**22.0**

**Source:** Authors own computation based on, Persons with Disabilities in India Survey: Schedule, 26 NSS 76^th^ Round (July 2018-December 2018)

**Table 3:** State-wise differentials in prevalence of disabilities per 1000 population.

State	Locomotor	Visual	Hearing	Speech and language	Mental retardation/ Intellectual disability	Mental illness	Other	Any disability
Jammu & Kashmir	7.3	1.9	1.3	1.8	1.1	1.2	0.3	14.8
Himachal Pradesh	12.0	1.8	2.3	1.5	2.5	1.6	0.2	21.9
Punjab	18.1	1.7	2.0	1.5	1.6	1.4	0.3	26.7
Chandigarh	6.2	0.2	1.6	0.9	1.5	0.0	0.4	10.8
Uttarakhand	7.7	2.5	2.2	1.4	1.1	1.4	0.2	16.3
Haryana	16.7	2.2	2.7	1.6	2.2	1.3	0.2	26.9
Delhi	7.7	1.0	1.0	0.8	1.0	0.9	0.3	12.7
Rajasthan	13.2	2.3	2.2	1.5	1.4	1.4	0.3	22.4
Uttar Pradesh	14.4	2.3	2.1	1.7	1.3	1.4	0.3	23.6
Bihar	9.6	1.5	1.2	2.1	1.2	0.8	0.2	16.5
Sikkim	5.0	5.3	5.4	5.3	0.6	0.6	0.1	22.4
Arunachal Pradesh	3.6	3.8	3.6	3.1	0.6	2.1	1.9	18.4
Nagaland	3.3	1.8	1.4	1.4	0.7	0.9	1.0	10.5
Manipur	3.3	0.7	1.2	1.0	0.8	0.6	0.4	8.1
Mizoram	2.2	1.4	2.0	2.8	1.8	0.7	0.1	10.8
Tripura	6.1	1.3	1.2	1.5	0.9	0.9	0.5	12.5
Meghalaya	3.3	1.5	1.7	1.6	1.0	0.4	0.1	9.7
Assam	8.3	3.0	2.8	1.8	0.8	1.0	0.3	17.9
West Bengal	10.3	2.1	1.9	2.3	1.7	1.8	1.2	21.3
Jharkhand	12.0	2.0	1.8	2.6	1.1	2.0	0.5	22.0
Odisha	16.0	3.7	4.6	2.5	2.0	2.2	1.4	32.5
Chhattisgarh	12.0	1.7	2.2	1.8	1.7	1.0	1.2	21.6
Madhya Pradesh	12.3	2.2	2.0	1.6	1.5	1.1	0.2	21.0
Gujarat	8.3	1.0	1.4	1.3	1.7	0.9	0.8	15.4
Daman & Diu	6.1	0.4	0.4	1.7	0.9	0.5	0.0	10.0
Dadra & Nagar Haveli	5.0	1.0	0.9	1.0	1.2	0.8	0.8	10.7
Maharashtra	13.5	1.9	2.0	1.6	1.7	1.0	0.7	22.4
Andhra Pradesh	16.7	2.4	3.8	2.6	2.0	1.0	0.9	29.5
Karnataka	12.9	3.4	3.1	1.9	1.7	0.8	0.1	24.0
Goa	6.8	0.8	2.0	0.9	1.4	1.1	0.4	13.4
Lakshadweep	12.7	2.3	1.7	0.6	2.1	0.6	0.0	20.0
Kerala	14.9	2.3	3.3	2.2	3.2	3.9	1.7	31.6
Tamil Nadu	9.9	2.0	2.6	1.8	1.6	0.9	0.6	19.4
Puducherry	13.7	2.8	4.7	1.4	1.9	1.7	0.0	26.2
Andaman & Nicobar Islands	8.2	1.1	1.0	1.6	1.3	1.3	0.5	15.1
Telangana	12.3	1.7	1.8	2.0	1.4	1.0	0.3	20.5
**Total**	**12.4**	**2.1**	**2.2**	**1.9**	**1.6**	**1.3**	**0.5**	**22.0**

**Source:** Authors own computation based on, Persons with Disabilities in India Survey: Schedule, 26 NSS 76^th^ Round (July 2018-December 2018)

### Out of pocket expenditure (OOPE)

OOPE for any disability was higher for males, those currently married, who belonged to other religion, and higher economic strata. OOPE among disabled was highest for those who suffered from other disability (INR, 3401), followed by speech and locomotor INR, 2942 and INR, 2675 respectively. Out of pocket expenditure due to health care for locomotor disability was higher among people who were working as a salaried employee/regular wage. Overall OOPE for disability-related health care amounted to INR, 2477 per person. Highest OOPE seen among those aged 15–35 years INR, 2809. OOPE increased as education level increased rising to INR, 3859 for those with secondary education and above (Table 4).

**Table 4:** Socioeconomic differentials in average out of pocket expenditure, household consumption expenditure, and share of out-of-pocket expenditure on total household expenditure for treatment of any disability in India.

Background Characteristics	Infrequent expenditure last 365 days			Usual monthly expenditure			Total OOPE Monthly			Share of out-of-pocket expenditure on total household expenditure	
	Medical expenditure	Nonmedical expenditure	OOPE	Medical expenditure	Nonmedical expenditure	OOPE	Medical expenditure	Nonmedical expenditure	OOPE	HCE	HCEB
**Age (in years)**											
0–14	9946	1526	11472	1256	310	1566	2085	437	2522	11950	21.1
15–35	15089	1643	16732	1129	286	1415	2387	422	2809	11553	24.3
36–59	11074	1351	12425	1217	271	1488	2140	384	2524	11754	21.5
60 and above	8718	1040	9758	1252	242	1494	1979	328	2307	12800	18.0
**Gender**											
Male	12600	1476	14076	1286	284	1570	2336	407	2743	11907	23.0
Female	8130	1055	9185	1146	243	1389	1823	331	2154	12538	17.2
**Education**											
Illiterate	7658	1026	8684	1020	221	1241	1658	307	1965	10800	18.2
Up to Primary	8819	1252	10071	1125	267	1392	1860	371	2231	11684	19.1
Middle	11830	1475	13305	1282	301	1583	2267	424	2691	12267	21.9
Secondary and above	18901	1831	20732	1784	348	2132	3359	500	3859	16018	24.1
**Marital Status**											
Never married	9170	1390	10560	1233	305	1538	1997	421	2418	12166	19.9
Currently married	12594	1379	13973	1268	267	1535	2317	382	2699	12267	22.0
Others	7359	941	8300	1102	217	1319	1716	295	2011	12040	16.7
**Relation to head of the household**											
Self/ Spouse of head	11334	1252	12586	1224	251	1475	2169	355	2524	11600	21.8
Married Child and spouse of child	15263	1889	17152	1340	318	1658	2612	476	3088	14038	22.0
Unmarried Child	8840	1345	10185	1177	290	1467	1913	402	2315	11294	20.5
Grand Child	11757	1879	13636	1472	375	1847	2452	532	2984	15473	19.3
Others family members and relatives	8697	1058	9755	1187	250	1437	1911	338	2249	14022	16.0
**Religion**											
Hindu	10937	1315	12252	1214	266	1480	2126	375	2501	11798	21.2
Muslim	8518	1156	9674	1234	258	1492	1944	354	2298	12486	18.4
Others	10625	1224	11849	1281	276	1557	2166	378	2544	15328	16.6
**Social group **											
SC/ST	8705	1166	9871	916	207	1123	1642	305	1947	9658	20.2
OBC	9439	1220	10659	1138	254	1392	1925	356	2281	11228	20.3
Others	13442	1459	14901	1560	323	1883	2680	445	3125	15321	20.4
**MPCE quintile **											
Poorest	5415	803	6218	635	151	786	1087	218	1305	4288	30.4
Poorer	10897	1140	12037	872	196	1068	1780	291	2071	7446	27.8
Middle	8845	1271	10116	1059	240	1299	1796	346	2142	10079	21.3
Richer	10477	1276	11753	1312	287	1599	2185	394	2579	13836	18.6
Richest	16742	1900	18642	2173	442	2615	3568	600	4168	24487	17.0
**Place of residence **											
Rural	9375	1200	10575	1001	235	1236	1782	335	2117	9563	22.1
Urban	12707	1438	14145	1614	319	1933	2673	439	3112	16827	18.5
**Type of Disability **											
Locomotor	12531	1388	13919	1260	256	1516	2304	371	2675	12208	21.9
Visual	5231	855	6086	668	192	860	1104	263	1367	10810	12.7
Hearing	5419	718	6137	687	191	878	1139	250	1389	12410	11.2
Speech	13675	1882	15557	1313	332	1645	2453	489	2942	12549	23.4
Mental Retardation	6268	1125	7393	1456	346	1802	1978	440	2418	13099	18.5
Mental Illness	6680	1090	7770	1187	280	1467	1744	371	2115	11699	18.1
Others	12514	1467	13981	1867	369	2236	2910	491	3401	13083	26.0
**Certificate of disability **											** **
No	11108	1283	12391	1240	253	1493	2165	360	2525	12264	20.6
Yes	8323	1298	9621	1150	319	1469	1843	427	2270	11881	19.1
**Percentage of disability**											** **
None	6683	1530	8213	919	266	1185	1476	394	1870	10248	18.3
40% or more but less than 60%	6627	1248	7875	1038	297	1335	1590	401	1991	11695	17.0
60% or more but less than 80%	9081	1297	10378	1179	327	1506	1936	435	2371	11825	20.1
80% or more	10324	1352	11676	1336	355	1691	2196	468	2664	12578	21.2
**Living Arrangements **											** **
Living with spouse and other household members	13589	1449	15038	1278	272	1550	2410	393	2803	13348	21.0
Living with spouse only	8870	1053	9923	1238	243	1481	1977	331	2308	7339	31.5
Living without spouse but with parents, children, other relatives, non-relatives	8597	1221	9818	1198	272	1470	1915	374	2289	12569	18.2
Living alone	3187	637	3824	725	129	854	991	182	1173	3994	29.4
**Total**	**10580**	**1286**	**11866**	**1223**	**265**	**1488**	**2104**	**373**	**2477**	**12192**	**20.3**

**Source:** Authors own computation based on, Persons with Disabilities in India Survey: Schedule, 26 NSS 76^th^ Round (July 2018-December 2018)

**Note:** OOPE = Out of pocket expenditure; MPCE = Monthly per capita consumption expenditure

### Share of out-of-pocket expenditure on total household expenditure

Overall, the Share of out-of-pocket expenditure on total household expenditure for disability-related health care amounted to 20.32%. Share of out-of-pocket expenditure on total household expenditure was higher among those aged 15–35 years (24.31%), those with of secondary education and above (24.9%), males (23.4%), those who are currently married (22%), lower economic strata (30.4%), rural residents (22.1%). Share of out-of-pocket expenditure on total household expenditure among people with any disability was highest in Lakshadweep followed by Uttarakhand and Andaman and Nicobar Islands (INR, 4648, 3984 and 3929 respectively). Health care burden was higher in Lakshadweep (29.5%) and Uttarakhand (29.3%) (Tables 4 and 5).

**Table 5:** State-wise differentials in average out of pocket expenditure, household consumption expenditure, and share of out-of-pocket expenditure on total household expenditure for treatment of any disability in India.

State	Infrequent expenditure last 365 days			Usual monthly expenditure			Total OOPE Monthly			Share of out-of-pocket expenditure on total household expenditure	
	Medical expenditure	Nonmedical expenditure	OOPE	Medical expenditure	Nonmedical expenditure	OOPE	Medical expenditure	Nonmedical expenditure	OOPE	HCE	HCEB
Jammu & Kashmir	6986	1636	8622	1246	353	1600	1828	490	2318	13065	17.7
Himachal Pradesh	10388	3293	13681	1496	514	2009	2361	788	3149	12403	25.4
Punjab	8294	922	9216	1151	204	1356	1843	281	2124	17941	11.8
Chandigarh	7400	2013	9413	1446	844	2290	2063	1011	3074	35542	8.7
Uttarakhand	19744	2122	21867	1789	373	2162	3435	550	3984	13573	29.4
Haryana	10984	1096	12079	1609	340	1950	2525	432	2956	19734	15.0
Delhi	12016	3122	15139	1847	590	2437	2848	850	3698	24304	15.2
Rajasthan	16679	2286	18965	1444	382	1825	2834	572	3406	14949	22.8
Uttar Pradesh	9996	1217	11213	1338	243	1581	2171	344	2515	10497	24.0
Bihar	4772	962	5734	535	134	669	933	214	1147	8303	13.8
Sikkim	5373	2167	7540	488	234	721	935	414	1350	10333	13.1
Arunachal Pradesh	1860	1309	3169	499	849	1348	654	958	1612	10106	16.0
Nagaland	17036	3390	20426	1590	131	1721	3010	413	3424	12748	26.9
Manipur	12825	5187	18012	565	669	1234	1634	1101	2735	10364	26.4
Mizoram	4093	1310	5403	614	162	776	955	271	1226	23827	5.2
Tripura	6917	1635	8552	996	224	1220	1573	360	1933	10301	18.8
Meghalaya	1571	1652	3223	343	255	598	474	392	866	11586	7.5
Assam	8546	1564	10111	861	207	1068	1573	338	1911	10758	17.8
West Bengal	6595	1023	7618	1294	250	1545	1844	336	2179	10953	19.9
Jharkhand	10125	1125	11251	1002	302	1304	1845	396	2242	10227	21.9
Odisha	6975	1002	7977	835	177	1012	1417	261	1677	7175	23.4
Chhattisgarh	11378	1382	12760	1206	181	1388	2155	297	2451	9407	26.1
Madhya Pradesh	10650	1797	12447	1244	328	1572	2132	478	2609	10798	24.2
Gujarat	10479	1120	11599	1222	305	1528	2095	399	2494	14876	16.8
Daman & Diu	12809	911	13720	988	213	1201	2055	289	2344	12637	18.6
Dadra & Nagar Haveli	1650	56	1706	1225	335	1561	1363	340	1703	10208	16.7
Maharashtra	18955	1423	20378	1324	270	1594	2904	388	3292	13308	24.7
Andhra Pradesh	9120	1099	10219	1340	219	1559	2100	311	2410	10496	23.0
Karnataka	11753	1002	12755	1023	238	1261	2002	322	2324	12511	18.6
Goa	4739	1618	6357	1412	557	1970	1807	692	2499	16782	14.9
Lakshadweep	21341	7291	28631	1545	717	2262	3323	1325	4648	15732	29.5
Kerala	9570	1162	10732	1406	335	1741	2204	431	2635	13650	19.3
Tamil Nadu	12063	1549	13612	926	271	1197	1931	400	2332	11100	21.0
Puducherry	9598	2918	12516	1811	584	2395	2611	827	3438	13113	26.2
Andaman & Nicobar Islands	16117	9211	25329	1453	366	1819	2796	1133	3929	20102	19.6
Telangana	10239	958	11197	1380	223	1603	2234	303	2536	11911	21.3
**India**	**10580**	**1286**	**11866**	**1223**	**265**	**1488**	**2104**	**373**	**2477**	**12192**	**20.3**

**Source:** Authors own computation based on, Persons with Disabilities in India Survey: Schedule, 26 NSS 76^th^ Round (July 2018-December 2018)

**Note:** OOPE = Out of pocket expenditure; MPCE = Monthly per capita consumption expenditure

### Catastrophic health expenditure (CHE)

Table 6 displays the households who faced the CHE due to health care expenditure on disabilities. About 57.1% and 34.1% of the household’s experiences to CHE due to OOPE on disability based on 10% and 20% thresholds respectively while 29.8% of the household’s health care expenditure exceeded PHCE of one household member, and in 13.2% households of two household members due to OOPE on disability. More households face CHE when a male was hospitalized at both the 10% (59.6% compared to 54.2% in females) and 20% threshold (36.7% compared to 31% for females). Proportionately more households in the rural areas faced CHE than those in urban areas (Table 6). More households were pushed to CHE 10% in Northern Chhattisgarh and Madhya Pradesh Central, whereas more households were pushed to CHE 20% in Northern Chhattisgarh (Table 7).

**Table 6:** Socioeconomic differentials in percentage of households pushed to catastrophic health expenditure due to incur the health care expenses on disability.

Background Characteristics	*First approach*		*Second approach*	
	10% threshold	20% threshold	PCCE*	TCCE**
**Age (in years)**				
0–14	58.5	34.7	36.0	16.2
15–35	57.9	35.3	33.2	16.0
36–59	57.7	34.8	28.9	12.9
60 and above	56.1	33.1	27.5	11.5
**Gender**				
Male	59.6	36.8	32.5	15.3
Female	54.3	31.0	26.6	10.7
**Education**				
Illiterate	55.3	31.8	28.5	12.2
Up to Primary	57.1	33.7	29.2	12.3
Middle	59.9	34.5	30.6	13.8
Secondary and above	59.9	39.7	33.1	16.2
**Marital Status**				
Never married	57.0	33.3	32.1	14.2
Currently married	59.4	36.5	31.5	14.6
Others	52.1	29.4	23.3	8.8
**Relation to head of the household**				
Self/ Spouse of head	59.0	36.2	27.8	12.2
Married Child and spouse of child	56.5	33.0	39.1	19.5
Unmarried Child	57.7	34.2	31.1	13.2
Grand Child	60.8	36.3	47.2	24.4
Others family members and relatives	49.7	26.9	28.8	12.2
**Religion**				
Hindu	58.1	35.4	30.4	13.6
Muslim	54.0	31.9	31.8	14.0
Others	53.6	25.6	20.6	8.2
**Social group **				
SC/ST	55.8	32.3	28.8	55.8
OBC	57.8	35.0	30.9	57.8
Others	57.3	34.4	29.2	57.3
**MPCE quintile **				
Poorest	62.3	35.9	39.4	19.1
Poorer	59.9	37.9	36.8	17.9
Middle	58.3	35.4	33.0	15.1
Richer	57.3	34.4	30.2	12.6
Richest	53.7	31.2	22.2	8.9
**Place of residence **				
Rural	59.6	35.8	31.6	14.5
Urban	52.8	31.1	26.7	10.9
**Type of Disability **				
Locomotor	59.3	36.2	31.0	14.2
Visual	41.0	21.8	17.8	6.9
Hearing	40.2	18.9	15.4	5.5
Speech	56.0	36.1	34.2	17.0
Mental Retardation	60.9	36.4	34.4	13.8
Mental Illness	59.5	32.5	30.3	12.3
Others	67.6	44.2	39.1	17.4
**Certificate of disability **				
No	57.6	34.2	30.2	13.7
Yes	55.1	33.5	28.2	11.1
**Percentage of disability**				
None	40.3	29.0	28.1	12.2
40% or more but less than 60%	52.3	31.5	26.5	9.7
60% or more but less than 80%	57.2	34.7	28.2	11.7
80% or more	59.0	35.8	31.0	12.4
**Living Arrangements **				
Living with spouse and other household members	56.0	33.0	34.1	16.3
Living with spouse only	73.1	50.3	18.1	5.3
Living without a spouse but with parents, children, other relatives, non-relatives	53.9	31.1	29.8	12.7
Living alone	75.7	49.0	4.5	0.5
**Total**	57.1	34.1	29.8	13.2

**Source:** Authors own computation based on, Persons with Disabilities in India Survey: Schedule, 26 NSS 76^th^ Round (July 2018-December 2018)

* = Household is pushed to CHE when the out-of-pocket expenditure exceeds the consumption expenditure of an average household member.

** = Household is pushed to CHE when the out-of-pocket expenditure exceeds the consumption expenditure of two household members.

**Table 7:** State-wise differentials in percentage of households pushed to catastrophic health expenditure due to incurred the health care expenses on disability.

State	*First approach*		*Second approach*	
	10% threshold	20% threshold	PCCE*	TCCE**
Jammu & Kashmir	47.3	24.3	25.4	12.6
Himachal Pradesh	71.7	49.7	40.4	18.1
Punjab	40.8	16.8	15.6	5.4
Chandigarh	35.0	15.4	12.0	6.5
Uttarakhand	63.2	43.5	42.9	30.6
Haryana	47.4	28.7	27.2	12.8
Delhi	45.1	23.9	20.0	7.9
Rajasthan	57.8	34.7	37.8	18.8
Uttar Pradesh	62.7	39.9	40.8	21.0
Bihar	40.8	21.4	21.4	8.4
Sikkim	55.4	19.9	14.5	2.9
Arunachal Pradesh	63.7	22.4	19.9	4.0
Nagaland	56.0	33.0	32.3	13.6
Manipur	53.3	35.9	35.3	17.7
Mizoram	13.0	4.5	3.8	1.5
Tripura	50.3	31.3	21.3	8.2
Meghalaya	19.6	8.6	9.6	2.2
Assam	47.0	26.4	26.0	11.7
West Bengal	55.7	33.6	26.6	10.3
Jharkhand	60.2	35.4	32.7	14.6
Odisha	65.6	39.5	32.6	12.9
Chhattisgarh	71.0	49.3	46.4	19.6
Madhya Pradesh	69.1	45.4	40.3	20.5
Gujarat	49.1	27.3	23.9	9.7
Daman & Diu	61.6	41.2	16.6	2.7
Dadra & Nagar Haveli	51.6	36.8	26.4	4.4
Maharashtra	61.2	37.7	33.6	16.1
Andhra Pradesh	64.8	38.9	24.5	8.8
Karnataka	52.2	29.9	25.8	11.0
Goa	41.6	26.9	14.0	4.7
Lakshadweep	77.7	53.5	65.4	40.2
Kerala	56.2	31.6	23.3	8.6
Tamil Nadu	56.3	37.6	28.8	8.7
Puducherry	40.4	14.4	13.3	10.8
Andaman & Nicobar Islands	48.9	23.0	23.0	11.9
Telangana	58.8	30.7	19.9	8.2
**India**	**57.1**	**34.1**	**29.8**	**13.2**

**Source:** Authors own computation based on, Persons with Disabilities in India Survey: Schedule, 26 NSS 76^th^ Round (July 2018-December 2018)

* = Household is pushed to CHE when the out-of-pocket expenditure exceeds the consumption expenditure of an average household member.

** = Household is pushed to CHE when the out-of-pocket expenditure exceeds the consumption expenditure of two household members.

### Poverty impact

Table 8 describes the households falling into poverty line (poverty headcount ratio) and average deficit from the poverty line (poverty gap ratio) due to OOP health payments for the treatment of disability. About 19% of the households fall below the poverty line due to treatment care expenditure for any disability and further significant differentials in the poverty gap ratio were observed across different socioeconomic characteristics. Higher proportion of households with disabled people in the age group 15–35 years, fall below the poverty line due to treatment care expenditure for any disability than others. Males, currently married, Hindu, Other backward classes, Rural, better-off households, regular wage/salaried employee had higher chances of falling to below the poverty line due to health care expenditure for disability. Impoverishment due to health care was highest in Lakshadweep, followed by Madhya Pradesh and Uttarakhand (Table 9).

**Table 8:** Socioeconomic differentials in households falling poverty line (poverty headcount ratio) and average deficit from the poverty line (poverty gap ratio) due to out-of-pocket health payments.

Background Characteristics	*Poverty Impact due to health care expenditure*	
	Poverty headcount ratio (%)	Poverty gap ratio (%)
**Age (in years)**		
0–14	19.6	8.2
15–35	21.0	16.6
36–59	19.9	12.1
60 and above	17.8	8.9
**Gender**		
Male	20.6	13.3
Female	17.4	8.2
**Education**		
Illiterate	19.2	8.7
Up to Primary	18.5	8.5
Middle	19.7	11.7
Secondary and above	19.4	18.7
**Marital Status**		
Never married	18.6	8.3
Currently married	20.4	12.7
Others	16.8	7.6
**Relation to head of the household**		
Self/ Spouse of head	19.9	13.2
Married Child and spouse of child	19.6	13.4
Unmarried Child	19.0	8.8
Grand Child	20.2	7.6
Others family members and relatives	16.5	6.5
**Religion**		
Hindu	20.0	11.8
Muslim	19.2	7.7
Others	10.9	8.3
**Social group **		
SC/ST	19.2	9.6
OBC	20.5	10.0
Others	17.4	13.2
**MPCE quintile **		
Poorest	0.0	0.0
Poorer	26.1	7.1
Middle	34.8	13.0
Richer	27.8	17.7
Richest	9.1	10.6
**Place of residence **		
Rural	21.1	12.0
Urban	15.6	9.1
**Type of Disability **		
Locomotor	20.2	13.2
Visual	12.9	5.0
Hearing	12.1	4.2
Speech	19.6	12.1
Mental Retardation	18.4	6.0
Mental Illness	20.7	7.7
Others	22.6	13.9
**Certificate of disability **		
No	19.4	11.4
Yes	17.9	9.1
**Percentage of disability**		
None	19.2	5.9
40% or more but less than 60%	15.9	6.9
60% or more but less than 80%	18.9	10.2
80% or more	19.7	11.6
**Living Arrangements **		
Living with spouse and other household members	19.3	12.2
Living with spouse only	24.1	21.0
Living without a spouse but with parents, children, other relatives, non-relatives	17.8	7.8
Living alone	22.8	14.4
**Total**	**19.1**	**11.0**

**Source:** Authors own computation based on, Persons with Disabilities in India Survey: Schedule, 26 NSS 76^th^ Round (July 2018-December 2018)

**Table 9:** State-wise differentials in households falling poverty line (poverty headcount ratio) and average deficit from the poverty line (poverty gap ratio) due to out-of-pocket health payments.

State	*Poverty Impact due to health care expenditure*	
	Poverty headcount ratio (%)	Poverty gap ratio (%)
Jammu & Kashmir	15.2	7.0
Himachal Pradesh	20.4	9.9
Punjab	6.2	3.4
Chandigarh	3.2	0.5
Uttarakhand	27.5	22.0
Haryana	16.2	8.2
Delhi	8.9	4.3
Rajasthan	22.4	17.0
Uttar Pradesh	24.2	11.6
Bihar	14.5	3.5
Sikkim	6.9	2.4
Arunachal Pradesh	6.4	11.3
Nagaland	26.0	18.5
Manipur	18.4	12.1
Mizoram	1.8	0.3
Tripura	15.5	6.2
Meghalaya	8.4	0.8
Assam	19.4	8.2
West Bengal	21.8	10.0
Jharkhand	20.1	9.4
Odisha	17.4	5.8
Chhattisgarh	22.2	10.3
Madhya Pradesh	27.2	12.5
Gujarat	13.7	9.1
Daman & Diu	5.7	7.3
Dadra & Nagar Haveli	24.1	3.7
Maharashtra	22.0	20.3
Andhra Pradesh	20.0	14.4
Karnataka	15.5	11.4
Goa	6.1	1.5
Lakshadweep	44.2	24.4
Kerala	12.9	8.9
Tamil Nadu	24.8	12.8
Puducherry	11.1	19.1
Andaman & Nicobar Islands	11.9	27.6
Telangana	20.1	9.9
**Total**	**19.1**	**11.0**

**Source:** Authors own computation based on, Persons with Disabilities in India Survey: Schedule, 26 NSS 76^th^ Round (July 2018-December 2018)

## Discussion

The present study examined the prevalence of disability and associated cost to treating the different types of disability in India. The present study also attempts to analysis the burden on households like income loss, catastrophic health care expenditure, and poverty impact due health care expenditure on disability by selected socioeconomic characteristics across the states of India using the recent national representative cross-sectional NSS 76^th^ round survey data which is specially focus on disability on the theme ‘Persons with Disabilities in India Survey. There are several notable findings.

The prevalence of any disability was found to be 22 per 1000 persons in India. Among them, 12.4 per 1000 person were found to be suffering from locomotor disability. Prevalence of visual, hearing and speech disability was 2.1, 2.2 and 1.9 per 1000 person respectively. Mental retardation and mental illness were found to be among 1.6 and 1.4 per 1000 persons. Similar finding has been indicated by census 2011 [41].

In India, limited studies talk about the financial burden due to disability. Overall, OOPE for disability-related health care amounted to INR, 2477 per person with the highest OOPE seen among those aged 15–35 years INR, 2809. OOPE increased as education level increased rising to INR, 3859 for those with secondary education and above. OOPE among disabled was highest for those who suffered from other disability (INR, 3401), followed by speech and locomotor INR, 2942 and 2675 respectively. Though the OOPE was more among higher economic stat group (INR, 4168) compared to poorest economic group (INR, 1305) but the percentage of health care burden was higher among the poorest (30.4%) compared the Richest (17.0%). Our study results relating to financial burden follow similar forms to pat study done by Plamer et al., 2011 and highlighted that poor households are more faced the health care burden on disability treatment [42]. OOPE among people with any disability was highest in Lakshadweep followed by Uttarakhand and Andaman and Nicobar Islands (INR, 4648, 3984 and 3929 respectively). Health care burden was higher in Lakshadweep (29.5%) and Uttarakhand (29.3%). Region wise, OOPE was highest in Lakshadweep (INR, 4648) and Share of out-of-pocket expenditure on total household expenditure was highest in Rajasthan Southern (34.7%).

When we talk about CHE due to OOPE, analysis indicates that about 57.1%, and 34.1% of the households enforced to CHE due to OOPE on disability based on 10% and 20% thresholds respectively, similar finding observed by past study which shows that households with disabled persons are at faced higher risk to the CHE and distress financing [42]. Various past studies examine the occurrence of CHE based on the ratio method that is ratio of OOPE on household consumption expenditure, which exceeds a certain threshold [43–47]. Even though using the ratio methods, the present study also explores the new method for examine the CHE, best on the authors information it is very new and justify method for CHE calculation if disparity in households’ size is exits and this method claimed that categorizing a household as experiencing CHE based on the ratio method doesn’t reflect the actual condition [20, 32]. The results show that 29.8% of the household’s faced CHE based on the average per capita consumption expenditure and in 13.2% households of faced CHE based on average consumption of two household members due to OOPE on disability. The level of CHE was fluctuating across socioeconomic characteristics, States, and type of types of disability. More households faced CHE if the person was currently married at both thresholds (59.3% and 36.5% at the 10% threshold and 20% threshold respectively). More than 70 percent of the households in Lakshadweep, Chhattisgarh and Himachal Pradesh were pushed to CHE at the 10% and 20% threshold. More households were pushed to CHE 10% in Northern Chhattisgarh and Madhya Pradesh Central, whereas more households were pushed to CHE 20% in Northern Chhattisgarh. Punjab had highest prevalence of locomotor disability (18 persons per 1000 followed by visual disability was highest in Sikkim (5.3 person per 1000), and mental retardation and mental illness was highest in the State of Kerala. Previous studies have also discussed geographical variations of the disease and disability [48].

Our study indicates that about 19% of the households fall below the poverty line due to treatment care expenditure for any disability. Further significant differentials in the poverty gap ratio were observed across different socioeconomic characteristics and type of disability. Higher proportion of households with disabled people in the age group 15–35 years, fall below the poverty line due to treatment care expenditure for any disability than others age group. The impoverishment effect among Males, currently married, Hindu, Other backward classes, Rural, had higher chances of falling to below the poverty line due to incurred OOPE on disability. Impoverishment was highest in Lakshadweep, followed by Madhya Pradesh and Uttarakhand due OOPE on disability.

### Strengths of the study

The strength of this study derived from its use of national representative cross-sectional from the NSS 76^th^ round based on the theme of Persons with Disabilities in India Survey among all the states of India which offers the generalizability to the study findings. This study analyzed indices, namely the health care burden, catastrophic expenditure and impoverishment using standard definitions.

### Limitations of the study

While, this study has numerous strengths, there are some limitations as well. Firstly, this study reports a cross-sectional survey data where recall bias is a major limitation for health care expenditure. Secondly, the survey did not collate data on indirect cost like opportunity costs, such as income losses due to disability and wage loss of caregivers. Thirdly, the survey not collected data on the household hardship financing for manage the cost on disability.

## Conclusion

Result from this study put forward significant insight that to avert catastrophic health care expenditure and poverty, proper interventions in terms of improving healthcare access and eliminating economic barriers to accomplish disability are essential and these interventions should target weaker section, such as households whose members are suffering from disabilities, households who belong the lowers economic Stata or households in the bottom socioeconomic status. Findings also confirm that socioeconomic inequalities in OOPE, catastrophic health care expenditure, hardship financing and poverty due to OOPE for disabilities exist in India, possibly due to poor utilization of healthcare service by poor households and socially weaker section, therefore, policymakers need to design policies that will make sure that resources for healthcare are equitably dispersed and benefit both the poor and rich. Out-of-pocket health expenditure due to disability in India has been established to be a progressive and unbalanced form of financing health system with catastrophic health expenditure and pushed household to poor from non-poor and effects the living standards of household too. The high Out-of-pocket health expenditure due to disability will affect to utilization and quality of healthcare facilities and household’s financial risk protection.

### Program implications

Findings from this study provide evidence for policies and financial support to people with disabilities to avoid medical impoverishment due to their health care expenditure on disability. It is important to highlight the fact that even after the introduction of government social health insurance schemes, there is high prevalence of catastrophic expenditure due to disability. In response to the escalating burden of disability nationwide, there is a pressing and immediate need to establish comprehensive healthcare services aimed at aiding those affected. The implementation of programs and policies dedicated to supporting individuals with disabilities must operate with a mission-driven approach, ensuring that assistance reaches even the most underserved segments of our society.

Therefore, there is a need to tailor disability-specific insurance schemes designed for disabled people. Although the Ayushman Bharat Yojana India’s social protection scheme, is markable step towards addressing the health needs of disadvantaged groups but still there is need for public and media outlets to embark on the advocacy to reduce out of pocket health care expenditure and demand for health system reforms from governments at the national and states levels. This study also recommends that developing appropriate policies to expand the effectiveness of health insurance, such as developing specific disability service package to be included in the health insurance program like Ayushman Bharat.

## References

[1] Saikia N, Moradhvaj Bora JK. Gender difference in health-care expenditure: Evidence from India human development survey. PLoS One. 2016;11(7):1–15. doi: 10.1371/journal.pone.0158332 27391322 PMC4938214

[2] Krahn Gloria, and Campbell Vincent A. Evolving views of disability and public health: the roles of advocacy and public health. Disability and health journal. 2011; 4(1):12–18. doi: 10.1016/j.dhjo.2010.05.005 21168802

[3] Narayan Choudhary Laxmi, and John Thomas. The Rights of Persons with Disabilities Act, 2016: Does it address the needs of the persons with mental illness and their families. Indian journal of psychiatry. 2017; 59(1): 17. doi: 10.4103/psychiatry.IndianJPsychiatry_75_17 28529356 PMC5419007

[4] Banurekha Velayutham, Kangusamy Boopathi, and Mehendale Sanjay. Prevalence of disability in Tamil Nadu, India. The National medical journal of India. 2017; 30(3): 125–130. 28936995

[5] Deon Filmer. Disability, poverty, and schooling in developing countries: results from 14 household surveys. The World Bank Economic Review. 2008; 22(1): 141–163.

[6] Kandamuthan Murugan, and Subodh Kandamuthan. The economic burden of disabled children on families in Kerala in South India. In iHEA 2007 6th World Congress: Explorations in Health Economics Paper, Centre for Development Studies Discussion Paper, no. 91. 2004.

[7] Singh Nirbhay N, Giulio E Lancioni, Alan SW Winton, Ashvind N Singh, Angela D Adkins, and Judy Singh. Clinical and benefit—cost outcomes of teaching a mindfulness-based procedure to adult offenders with intellectual disabilities. Behavior Modification. 2008; 32(5): 622–637. doi: 10.1177/0145445508315854 18362201

[8] Harriss-White, Barbara. On to a loser: Disability in India. B. Harriss-White and S. Subramaniam (eds) 1999; 135–59.

[9] Leibson Cynthia L., Katusic Slavica K., Barbaresi William J., Ransom Jeanine, and Peter C. O’Brien. Use and costs of medical care for children and adolescents with and without attention-deficit/hyperactivity disorder. Jama. 2001; 285(1): 60–66. doi: 10.1001/jama.285.1.60 11150110

[10] Swensen Andrine R., Howard G Birnbaum, Kristina Secnik, Maryna Marynchenko, Paul Greenberg, and Claxton A M I. Attention-deficit/hyperactivity disorder: increased costs for patients and their families. Journal of the American Academy of Child & Adolescent Psychiatry. 2003; 42(12): 1415–1423. doi: 10.1097/00004583-200312000-00008 14627876

[11] Goetzel Ron Z, Stacey R Long, Ronald J Ozminkowski, Kevin Hawkins, Shaohung Wang, and Wendy Lynch. Health, absence, disability, and presenteeism cost estimates of certain physical and mental health conditions affecting US employers. Journal of occupational and environmental medicine. 2004; 398–412. 15076658 10.1097/01.jom.0000121151.40413.bd

[12] Montgomery Kyle, Ryan Timothy J Andrew Krause, and Starkey Chad. Assessment of athletic health care facility surfaces for MRSA in the secondary school setting. Journal of Environmental Health. 2010; 72(6): 8–11. 20104827

[13] Mitchell Rebecca J, and Paul Bates. Measuring health-related productivity loss. Population health management 14, no. 2 (2011): 93–98. doi: 10.1089/pop.2010.0014 21091370 PMC3128441

[14] Sen Amartya. Human rights and capabilities. Journal of human development. 2005; 6(2): 151–166.

[15] Raut Lakshmi K, Manoranjan Pal, and Premananda Bharati The economic burden of disability in India: Estimates from the NSS data. Available at SSRN 2432546. 2014.

[16] National Sample Survey Organisation (NSSO) *Persons with Disabilities in India*. Government of India, Ministry of Statistics and Programme Implementation, National Statistical Office. 2018 Report No. 583.

[17] Yadav J, Allarakha S, John D, Menon G, Venkateswaran C, Singh R. Catastrophic Health Expenditure and Poverty Impact Due to Mental Illness in India. Journal of Health Management. 2023; 25(1):8–21

[18] Davoudi-Kiakalayeh Ali, Dalal Koustuv, Shahrokh Yousefzade-Chabok Bjarne Jansson, and Mohammadi Reza. Costs related to drowning and near drowning in northern Iran (Guilan province). Ocean & coastal management. 2011; 54(3): 250–255.

[19] Barnsley Paul D, Amy E Peden, and Justin Scarr. Calculating the economic burden of fatal drowning in Australia. Journal of safety research. 2018; 67: 57–63. doi: 10.1016/j.jsr.2018.09.002 30553430

[20] Yadav J, Menon GR, & John D. Disease-specific out-of-pocket payments, catastrophic health expenditure and impoverishment effects in India: an analysis of National Health Survey data. Applied Health Economics and Health Policy. 2021; 19, 769–782. doi: 10.1007/s40258-021-00641-9 33615417

[21] Yadav J, John D, Menon G. Out of pocket expenditure on tuberculosis in India: Do households face hardship financing? Indian J Tuberc. 2019;66(4):448–460. doi: 10.1016/j.ijtb.2019.02.016 31813431

[22] Mitra S, Findley PA, Sambamoorthi U. Health care expenditures of living with a disability: Total expenditures, out-of-pocket expenses, and burden, 1996 to 2004. Arch. Phys. Med. Rehabil. 2009; 90:1532–1540. doi: 10.1016/j.apmr.2009.02.020 19735781

[23] Sahoo AK, Madheswaran S. Socio-economic Disparities in Health Care Seeking Behaviour, Health Expenditure and Its Source of Financing in Orissa. J Health Manag. 2014;16(3):397–414. doi: 10.1177/0972063414539614

[24] Shetty BS, Shetty M. Epidemiology of drowning in Mangalore, a coastal Taluk of South India. Journal of Forensic and Legal Medicine. 2007 Oct 1;14(7):410–5. doi: 10.1016/j.jflm.2007.01.004 17720592

[25] Jeetendra Yadav, John Denny, Geetha R Menon, Richard C Franklin, and Amy E Peden. Nonfatal drowning-related hospitalizations and associated healthcare expenditure in India: An analysis of nationally representative survey data. Journal of safety research. 2022; 82: 283–292. doi: 10.1016/j.jsr.2022.06.003 36031256

[26] Caroline Lukaszyk, Mittal Srabani, Gupta Medhavi, Das Rumeli, Ivers Rebecca, and Jagnoor Jagnoor. The impact and understanding of childhood drowning by a community in West Bengal, India, and the suggested preventive measures. Acta paediatrica. 2019; 108(4):731–739. doi: 10.1111/apa.14592 30252948

[27] Mohanty Sanjay K, and Kastor Anshul. Out-of-pocket expenditure and catastrophic health spending on maternal care in public and private health centres in India: a comparative study of pre and post national health mission period. Health Economics Review. 2017;7: 1–15. 28921477 10.1186/s13561-017-0167-1 PMC5603466

[28] Sunil Rajpal, Kumar Abhishek, and Joe William. Economic burden of cancer in India: Evidence from cross-sectional nationally representative household survey, 2014. PloS ONE. 2018; 13(2): e0193320. doi: 10.1371/journal.pone.0193320 29481563 PMC5826535

[29] Soumitra Ghosh. Catastrophic payments and impoverishment due to out-of-pocket health spending. Economic and Political Weekly. 2011; 63–70.

[30] Sakthivel Selvaraj, Farooqui Habib Hasan, and Karan Anup. Quantifying the financial burden of households’ out-of-pocket payments on medicines in India: a repeated cross-sectional analysis of National Sample Survey data, 1994–2014. BMJ open. 2018; 8(5):e018020. doi: 10.1136/bmjopen-2017-018020 29858403 PMC5988077

[31] Kumar K, Singh A, Kumar S, et al. Socio-Economic Differentials in Impoverishment Effects of Out-of-Pocket Health Expenditure in China and India: Evidence from WHO SAGE. Nugent RA, ed. PLoS One. 2015;10(8):e0135051. doi: 10.1371/journal.pone.0135051 26270049 PMC4536075

[32] Joe W, Rajpal S. Unravelling the socioeconomic gradient in the incidence of catastrophic health care expenditure: A comment. Health Policy Plan. 2018;33(5):699–701. doi: 10.1093/heapol/czy026 29617995

[33] Wagstaff A, Van Doorslaer E. Catastrophe and impoverishment in paying for health care: With applications to Vietnam 1993–1998. Health Econ. 2003;12(11):921–933. doi: 10.1002/hec.776 14601155

[34] Raveendran G. A Review of Rangarajan Committee Report on Poverty Estimation: http://dx.doi.org/101177/0973703016648033. Published online July 14, 2016. doi: 10.1177/0973703016648033

[35] All India Consumer Price Index (AICPI), 2020 cyberjournalist.org.in, http://cyberjournalist.org.in/manisana/aicpi.html. Accessed 27 Apr. 2022.

[36] Bajwala VR, John D, Rajasekar TD, Murhekar MV. Severity and costs associated with hospitalization for dengue in public and private hospitals of Surat city, Gujarat, India, 2017–2018. Transactions of the Royal Society of Tropical Medicine and Hygiene. 2019; 113(11):661–669 doi: 10.1093/trstmh/trz057 31294808

[37] McDonald KE, Balcazar FE, Keys CB. Youth with Disabilities. *Handb Youth Mentor*. 2012;29(2011):493–507. doi: 10.4135/9781412976664.n33

[38] CDC. Disability and Health—People with Disabilities | CDC. Centers for Disease Control and Prevention. 2020, www.cdc.gov, https://www.cdc.gov/ncbddd/disabilityandhealth/people.html

[39] StataCorp. Stata Statistical Software: Release 13. 2013; College Station, TX: StataCorp LP.

[40] Korn Edward L, and Barry I Graubard. Simultaneous testing of regression coefficients with complex survey data: Use of Bonferroni t statistics. The American Statistician. 1990; 44(4): 270–276.

[41] Census of India Office of the Registrar General & Census Commissioner, India 2011.

[42] Plamer M, Nguyen T, Neeman T, Berry H, Hull T, Harley D. Health care utilization, cost burden and coping strategies by disability status: an analysis of the Vietnam National Health Survey. The International journal of health planning and management. 2011;26(3):e151–e168. doi: 10.1002/hpm.1052 20583316

[43] Van Doorslaer E, O’Donnell O, Rannan‐Eliya RP, Somanathan A, Adhikari SR, Garg CC, et al. Catastrophic payments for health care in Asia. Health economics. 2007; 16(11), 1159–1184. doi: 10.1002/hec.1209 17311356

[44] Mataria A, Raad F, Abu-Zaineh M, & Donaldson C. Catastrophic healthcare payments and impoverishment in the occupied Palestinian territory. Applied Health Economics and Health Policy. 2010; 8: 393–405. doi: 10.2165/11318200-000000000-00000 21043541

[45] Yadav J, John D, Menon GR, Franklin RC, & Peden AE. Nonfatal drowning-related hospitalizations and associated healthcare expenditure in India: An analysis of nationally representative survey data. Journal of safety research. 2022; 82, 283–292. doi: 10.1016/j.jsr.2022.06.003 36031256

[46] Somkotra T, Lagrada LP. Which households are at risk of catastrophic health spending: Experience in Thailand after universal coverage. Health Aff. 2009;28(3). doi: 10.1377/hlthaff.28.3.w467 19336470

[47] Yadav J, Devi S, Singh MN, & Manchanda N. Health care utilization and expenditure inequities in India: Benefit incidence analysis. Clinical Epidemiology and Global Health. 2022; 15, 101053.

[48] Nilima N, Puranik A, Kaushik S, et al. Geographic variation and factors associated with breast cancer screening in India using a spatial durbin approach. Spatial Demography. 2023;11(3). doi: 10.1007/s40980-023-00114-8

